# Integration of a nationally procured electronic health record system into user work practices

**DOI:** 10.1186/1472-6947-12-15

**Published:** 2012-03-08

**Authors:** Kathrin M Cresswell, Allison Worth, Aziz Sheikh

**Affiliations:** 1 eHealth Research Group, Centre for Population Health Sciences, The University of Edinburgh, Scotland, UK

## Abstract

**Background:**

Evidence suggests that many small- and medium-scale Electronic Health Record (EHR) implementations encounter problems, these often stemming from users' difficulties in accommodating the new technology into their work practices. There is the possibility that these challenges may be exacerbated in the context of the larger-scale, more standardised, implementation strategies now being pursued as part of major national modernisation initiatives. We sought to understand how England's centrally procured and delivered EHR software was integrated within the work practices of users in selected secondary and specialist care settings.

**Methods:**

We conducted a qualitative longitudinal case study-based investigation drawing on sociotechnical theory in three purposefully selected sites implementing early functionality of a nationally procured EHR system. The complete dataset comprised semi-structured interview data from a total of 66 different participants, 38.5 hours of non-participant observation of use of the software in context, accompanying researcher field notes, and hospital documents (including project initiation and lessons learnt reports). Transcribed data were analysed thematically using a combination of deductive and inductive approaches, and drawing on NVivo8 software to facilitate coding.

**Results:**

The nationally led "top-down" implementation and the associated focus on interoperability limited the opportunity to customise software to local needs. Lack of system usability led users to employ a range of workarounds unanticipated by management to compensate for the perceived shortcomings of the system. These had a number of knock-on effects relating to the nature of collaborative work, patterns of communication, the timeliness and availability of records (including paper) and the ability for hospital management to monitor organisational performance.

**Conclusions:**

This work has highlighted the importance of addressing potentially adverse unintended consequences of workarounds associated with the introduction of EHRs. This can be achieved with customisation, which is inevitably somewhat restricted in the context of attempts to implement national solutions. The tensions and potential trade-offs between achieving large-scale interoperability and local requirements is likely to be the subject of continuous debate in England and beyond with no easy answers in sight.

## Background

The literature is littered with examples of "failed" electronic health record (EHR) implementations in healthcare settings, and particularly large-scale ones although these are, in the light of the significant anticipated benefits, increasingly pursued internationally [1-6]. A recurring theme in this literature is that of clinicians finding it difficult to integrate the use of information technology (IT) systems into their work practices and care provision [6-20]. As a result, end-users may either partially use (i.e. only use the parts that they perceive as useful), develop "workarounds" (which we define as behaviour employed by users to overcome a perceived limitation in a technical system), or avoid using the system altogether ("non-compliance") [21-23]. These coping strategies may in turn lead to the technology being used in ways other than originally intended or not being used at all, which can result in important disturbances to organisational functioning and worse still compromise patient safety [24].

This literature indicates that difficulty integrating technology with the work practices of healthcare staff is particularly likely in situations in which a new system is perceived as being imposed on users [21]. Integration of EHRs with user work practices is therefore a central concern for large-scale national ventures where system design often reflects a focus on interoperability (i.e. the ability meaningfully to exchange information between systems). A key challenge then is to design a system that is able to deal with the potentially multiple (at times conflicting) requirements of different stakeholders and within different user groups in an environment that is characterised by complexity and variability [19,25]. It is however also important to use the technology to facilitate change and to implement an (agreed) change management programme on the back of this, rather than seeking simply to replicate existing processes.

As part of its national strategy, England procured three commercial EHR systems to be implemented in hospitals. These included Cerner Millennium, Lorenzo Regional Care (henceforth referred to as Lorenzo), and RiO. Lorenzo (described in Table 1) was planned to be implemented across the North, Midlands and Eastern region of England and was intended to be used in 219 hospitals. Our study drew on a subset of data obtained as part of a national evaluation of EHRs in English hospitals [26-28]. However, the focus of this work was a more in-depth exploration of the consequences of Lorenzo on both individual and collective work practices and stakeholder relationships. Our rationale for focusing on this particular type of software was that the system was implemented in consecutive releases, gradually replacing paper systems, which meant that changes in work practices could be traced over time and in more detail than would have been possible with a more rapid roll-out.

**Table 1:** Description of the properties of the nationally procured type of software that was the focus of our investigation

Lorenzo Regional Care	
	Developed by iSOFT in India and implemented by the Computer Sciences Corporation as part of England's national strategy

	The system was developed as it was being implemented and had releases with increasing capabilities that were implemented consecutively

	In the initial release, the system and paper processes were run in parallel as system capabilities were limited including clinical notes and requesting

	The second release replaced the existing Patient Administration system but this was only achieved in one hospital during our research

This work was informed by socio-technical principles and drew on Actor-Network Theory (ANT) [29,30], which helped us to investigate how the centrally procured EHR software has played an active role in shaping social relationships. This fluidity and interrelated nature of social and technical processes is often neglected in the technological determinism that typically dominates IT implementation strategies and views humans as passive recipients of technological innovation [31]. We therefore explored how the new technology has shaped professional practice and what consequences these changes had for organisational functioning, record keeping and patient care.

## Methods

### Design

We conducted a longitudinal comparative case study undertaking field work between February 2009 and November 2010. The majority of data were collected in three "early adopter" National Health Service (NHS) specialist care providers (henceforth referred to as 'sites') implementing early releases of Lorenzo in England. We conceptualised these as case studies to take into account local contingencies [29,30,32-35]. Our focus was on exploring the micro-environment of implementation and adoption, but without neglecting the wider implementation context (i.e. the national implementation environment), which was found to impact heavily on local developments [27,28].

### Sampling

We purposefully sampled sites from among the first few to implement Lorenzo software. Sampling criteria included the range of different settings (e.g. mental health, community, acute) and the timeframes of implementation (i.e. it had to begin during our study period).

Within each case study site, implementation team members were initially approached for interviews and observations through the local head/director of IT. We then used snowball sampling, asking individual participants to recommend further interviewees, actively seeking a range of different viewpoints. Individual users were initially recruited with the help of IT managers, ward managers and heads of service (again asking for recommendations of further interviewees). In doing so, we sampled a range of staff groups including both junior and senior nurses and doctors, allied health professionals and administrative staff.

### Data collection

As work practices are heavily context dependent, we focused on collecting qualitative data through interviews, observations and documents in the three selected sites. These data collection approaches helped us to gain an insight into local contingencies, as well as more general processes across settings and over time.

We conducted semi-structured interviews with users (i.e. those who were expected to use the technology within their everyday work including doctors, nurses, allied health professionals and administrative staff) and other hospital staff (e.g. managers, IT staff and clinical leads) in order to gain an insight into their experiences of using the new system. Particular issues explored included views and attitudes relating to the system and implementation strategy (if relevant over time), perceived challenges and changes to individual and collaborative working, and potential suggestions for improvements (see Table 2 for an indicative topic guide). The wide range of interviewees sampled allowed us to gain an insight into the consequences for individuals as well as for wider organisational functioning, which is dependent on effective collaborative working. The first round of interviews (Time 1) was conducted during early use, when sites had implemented the technology for between three months and one year. Where possible, interviews were conducted longitudinally, speaking to the same stakeholders around six months later (Time 2) in order to trace potential changes in attitudes and investigate the effects of more embedded use of the system. This temporal component also helped us to assess the local consequences of the continuously changing political and economic climate. This was particularly important in the context of the national implementation, as changes in national arrangements often had significant consequences for hospital staff. These included, for example, delays in implementation timelines (or conversely pressures to implement), and changes in strategic direction such as reduced software functionality [27].

**Table 2:** Sample topic guide employed in interviews

Questions	Can you tell me what you use Lorenzo for and how it contributes to patient care?
	What were your expectations before Lorenzo was put into use and were they fulfilled?

	How disruptive is the associated organisational change, for example in terms of learning new routines, new staff recruited, and needing to familiarise yourself with new practices?

	Has your behaviour/practice changed as a result of the introduction of Lorenzo? If so, in what way? Are there any unexpected changes to how you do things now?

	Do you see Lorenzo influencing your working style as part of a team or as a professional? (Prompt: e.g. in the way you communicate and collaborate with other health professionals and communicate with patients)?

	Can you tell me what, if any, might be the main benefits to you in your role from using Lorenzo? Do you see these benefits now? Are there any clear drawbacks in performing your role?

	Do you have any concerns about the introduction of Lorenzo? Can you tell me what these are?

	Are there any tasks or aspects of care that you feel will become more difficult or worse with the introduction of Lorenzo?

	Are there any changes that you would like to see made in how Lorenzo works? How could it be improved to be more acceptable and more effective in supporting care?

	What, if anything, would you miss most about Lorenzo if it were withdrawn?

	Did you have any problems when you first started using the system? How were these resolved?

	Do you have sufficient skills now to use Lorenzo to the maximum benefit?

	In what ways do you think Lorenzo will be/is a) better and b) worse than the system(s) it replaces? Why? (Probe: how did the 'old' one look-paper or mix of paper and electronic)?

In order to gain an insight into the more informal work practices of users, interviews were complemented by a mixture of object/activity/person-oriented observations of the software in use with on-site questioning, where appropriate and convenient. This involved "following the thing" (i.e. the software) and the various ways in which it was used and talked about in the clinical setting (e.g. by observing activities that involved the software) [36].

Hospital-specific documents, including project initiation documents, 'lessons learned' documents and local evaluation reports, were also collected in order to investigate the more formal (i.e. official or desired by management) changes to work practices. These included documents drafted before the system was implemented, outlining planned changes to work practices. We used these as a comparator to actual observed changes.

In order to examine concurrent developments in the wider political and economic landscape, we conducted additional semi-structured interviews with stakeholders outside sites. Interviewees here included governmental stakeholders, developers and representatives from the independent sector.

### Data handling and analysis

Transcribed interviews, observations, researcher field notes and documents were coded with the help of NVivo8 software [37], drawing on both deductive and inductive coding approaches [38]. We began by building a coding framework based on the existing literature surrounding technology implementation and associated challenges with integration into user work practices [6]. This formed the deductive part of our analysis and included dimensions of integration of the system with professional responsibilities, existing roles and routines, and changes in individual and collaborative working. The inductive part of the analysis involved recording of new themes emerging from the data. Evolving categories from these themes were fed back into subsequent data collection. In investigating how exactly the software shaped both individual and collective work practices (i.e. the micro-context), drawing on ANT proved particularly useful [39,40]. In doing so, our focus was on the active impact of the new technology on work practices of users, as it was beginning to replace established paper processes in the organisations. These insights were then used to draw conclusions about the wider environment in which these practices were situated by a constant process of "zooming in" and "zooming out" [39,40]. This approach helped us to build on the literature and gain a holistic picture of the implementation and adoption landscape whilst still allowing new themes to emerge. As the focus of the study was on exploring changes to user work practices, this category was investigated in more detail first within and then across cases and data sources as well as time points [41]. Triangulation was facilitated by using the query function in NVivo8, which allowed extracting data from different data sources and collection times, and drawing up thematic tables recording emerging issues in each data source, time-point, group of interviewee, and individual location.

We discussed findings in regular team meetings and kept a research journal capturing our emerging understanding of the cases. During the analysis process, we paid particular attention to reflecting on our own assumptions and the way we had reached our interpretations, seeking potential alternative explanations. This was achieved by testing whether these better explained the data, ensuring that findings did not occur due to chance, searching for evidence that may refute explanations, and by following up unexpected findings [41].

### Ethics

The observational component of this study received ethical approval from the East London and the City Research Ethics Committee on the 2nd of April 2009. The interview component submitted for ethical review to the same ethics committee but was classed as a service evaluation on the 9th of October 2008 (08/H0703/112). To protect the anonymity of participants and Trusts, we have removed potential identifiers wherever possible.

## Results

We collected data through interviews with 66 users and other hospital staff, carried out 38.5 hours of observations, collected 13 hospital-specific documents, and conducted interviews with 14 stakeholders outside the immediate hospital environment. A summary of data collected in each case study site is provided in Table 3. This also shows a breakdown of the number of different participating professions.

**Table 3:** Summary of data collected at each hospital

A large-scale implementation in an acute setting	A small-scale implementation in a community setting	A medium-scale implementation in a mental health setting	Overarching
- 41 interviews with 27 different interviewees (six implementation team members including clinical leads, managers and training professionals; 21 users including ward managers, consultants, nurses, ward clerks, administrative staff, pharmacists, and junior doctors) - 10 hours of observations - 13 pages of researcher field notes - Three hospital documents	- 26 interviews with 19 different interviewees (five implementation team members including clinical leads and managers; 14 users consisting of allied health professionals) - 24 hours of observations - Six pages of researcher field notes - Five hospital documents	- 21 interviews with 20 different interviewees (six implementation team members including clinical leads and managers; 14 users including doctors, nurses, psychologists, social workers, therapists, and administrative staff) - 4.5 hours of observations - 15 pages of researcher field notes - Four hospital documents	14 interviews with policy makers, system developers, and commercial sector representatives

As a result of our inductive analysis, we were able to identify a number of important factors relating to the integration of Lorenzo with user work practices. These are outlined in detail in Table 4 and included the following main categories:

• Software characteristics and associated consequences

• Coping strategies employed by software users in different contexts

• Direct and indirect knock-on effects.

**Table 4:** Emerging themes from our study

Software characteristics and their consequences	Design did not reflect reality of clinical practice
	Lack of customizability

	Perceived lack of fitness for purpose and lack of usability resulted in increased workloads for users

	Implementation strategy soft: initially parallel use of paper: intended workarounds

Coping strategies by users in different contexts	Some more powerful users resisted use

	Embedding of the system over time in smaller scale implementations that allowed intensive user involvement in software design

	Users who could not avoid using the system devised various ways to compensate for the increasing demands on their time and perceived shortcomings of the technology

	Often workarounds were unintended by management

Direct and indirect knock-on effects	Collaborative working-hierarchical structures and communication

	Time spend with patients and quality of interactions

	Paper: more distributed across geographical locations

	Managerial outputs became unpredictable often not reflecting the reality of what actually happened

	The medical record itself-delayed data entry

We elaborate on these themes below with supporting illustrative data (further data are available on request from the corresponding author).

### Software characteristics and associated consequences

The software in question was nationally procured, which meant that organisations, although to some extent free to choose which 'releases' (i.e. versions) they wished to implement, were presented with a fixed set of core releases that allowed limited customisability at either an organisational or group/individual user level. This reflected the then government's central desire to see interoperability. This was seen to be particularly important in the context of a state-run *national *health service. However, the focus on interoperability resulted in poor alignment between local work processes and software specifications. Users thus frequently expressed concern that the software lacked fitness for purpose and/or usability.

*"Two fundamental criticisms remain that the system is not, and what you see on the screen is not intuitive, in other words if you haven't been taught, haven't used it regularly enough to remember what all the sequences are, so if you're only dipping into it occasionally it is actually very, very difficult because it is, you cannot sort of automatically think well this is what I want to do next and look at it and say that's what I do ... the other criticism of it is the speed of the system that you don't, when you expect to move from one field to another it is not instant and that is a big concern in a system where one feels instinctively that it ought to be." *(Interview, Consultant, Time 2)

The limited ability to customise the software meant that it was difficult to adapt to suit local needs, which often resulted in significant increases in users' workloads.

*"Somebody rang me, 5-10 minutes on the phone, and it takes you, one time it took me over 50 minutes to do it all because it's a phone call, going downstairs to get the file cause you still have to have the file, finding the file, bringing that up then logging on, waiting, cause I just did this one thing and then putting it all on, trying to print it and it took 50 minutes and that was just a parent had rang me to say that they needed to cancel the session (laughs)." *(Interview, Therapist, Time 2)

The nature of Lorenzo also meant that it had to be implemented in phased releases as increasing functionality became available. Consequently, paper and electronic systems had to be run in parallel initially in order to compensate for the limited functionality of the early releases. Acknowledging that this temporary arrangement may have increased workload for end-users yet further, local implementation teams suggested possible workarounds for users to cope with these parallel systems. These, for example, included the:

"...*printing of Lorenzo notes and attaching these to paper files, creating written notes if there are issues with electronic notes (i.e. paper used as fallback)"*. (Source: local Deployment Verification Report)

### Coping strategies employed by software users in different contexts

We in addition observed a number of more unintended workarounds and coping strategies employed by users struggling to accommodate software that was perceived to be of poor usability. Some users, particularly those with more autonomy such as senior consultants, resisted use altogether by insisting on using paper records.

*"... medical staff sort of dig their heels in and then don't do it, do they and if they can get out of doing it they'll do it on paper..." *(Interview, Administrative Staff, Time 2).

Others, notably those who could not avoid using the system, devised various ways to compensate for the increasing demands on their time and concerns about the shortcomings of the technology. The most commonly employed techniques here included using other systems to compensate (e.g. typing letters in Microsoft Word as the spell-check was perceived to be much faster than that embedded in the software and/or reverting to paper systems); partially using the system (e.g. not recording certain activities if they were not viewed as important); and using the system in ways other than intended by management (e.g. getting around compulsory boxes by cross-referencing).

*"So you end up, because you can't cut and paste so you can't say look OK this is the same let's cut and paste it into here, you end up having to write it multiple, multiple times or you end up having to cross-reference and I actually think it is safer for the [patient] to have all the risk stuff clearly and concisely written in one place. The nature of the form means that one place isn't the form so I actually write it very clearly in the progress note or the assessment form and I always in the boxes that open up I just put 'please refer to [name of form]', which then increases the amount that the risk indicator is a tick box exercise." *(Interview, Nurse, Time 2)

Over time, some of these difficulties of integrating Lorenzo within users' everyday work practices attenuated to some extent as they became more familiar with the system. This was particularly true in smaller-scale deployments in sites that had invested significant time and resources to adapt the software to fit with their everyday practices. In addition to more effective integration with work practices as a consequence of increased familiarity, stakeholders reported at follow-up interviews that, as they received system upgrades, technical performance had improved significantly, particularly in relation to speed.

*"I think the speed difference is massive from when we first started to now, you hardly wait at all... A lot has changed... I know the steps to take to find stuff so it's not a problem to find stuff." *(Interview, Allied Healthcare Professional, Time 2)

In addition, some of the interim workarounds, such as printing forms completed on Lorenzo and attaching these to paper files, attenuated when the whole service used Lorenzo. Over time, paper appeared to progressively lose its significance, increasingly being used as a *"back-up system"*, with users employing compensating techniques such as, for example, performing another activity whilst waiting for the system (such as switching on the computer and making a cup of coffee); inputting less descriptive data as this helped to speed up data entry; or allocating extra time at the end of consultations for correcting spelling mistakes made whilst typing.

### Direct and indirect knock-on effects

Coping strategies employed by users had several direct and indirect knock-on effects. These are considered below in relation to collaborative working, patients, paper records, managerial outputs and recording activity.

#### Collaborative working-hierarchical structures and communication

The new technology impacted on the ways in which the healthcare team interacted. This was in some instances seen as a positive consequence, but in other situations as a negative development. In relation to the former, some users felt that using the system helped to make communication more effective over longer distances.

*"Yeah it is [more effective] because now we know that definitely everybody has access so things like, so last week we had a really urgent nail surgery on the Wednesday and I could actually book her in to have it on the Thursday knowing safely that her assessment was all there and I didn't have to rush off a set of notes and everything else it was all done. And that's only minor benefits for us but everyday where we use it more and more now we're paper free we just think of more things." *(Interview, Allied Healthcare Professional, Time 2)

On the other hand, users also reported that the system changed the way the healthcare team interacted in negative ways. This was mainly expressed in relation to changing professional roles and responsibilities with an increased emphasis on administrative tasks. It was seen as particularly problematic by clinical staff who expressed concern that the resulting displacement of administrative duties on to their shoulders was detracting from their more pressing clinical responsibilities.

*"It takes you much, much, much, much, much longer; it doesn't really help us at the moment. This version is absolutely useless to be honest and yeah, it's a waste of time, you're sitting in front of a computer and you should see basically patients and doing something and so instead of this you're typing in something, and you're kind of frustrated when you wait for something that took half a minute as a chest X-ray, it takes now at least 10 minutes..." *(Interview, Junior Doctor, Time 1).

Similarly, the system tended to change professional responsibilities, often making existing hierarchies more visible. For example, it only allowed nurses who had special training to order X-rays. Other nurses had to ask the doctors to order these if necessary. In such cases, doctors then had to complete the form, which created more (unwelcome) work for them. Conversely, nurses felt that some of their professional autonomy had been eroded (although it has to be noted that officially most nurses were not allowed to order X-rays-they were doing it informally by completing the cards on behalf of a doctor).

*"... for example you get a load of new doctors on the ward and you get a patient in with a specific condition you know automatically before the consultant even comes round what investigations that patient is to have, because there's a protocol so you know what they're going to have or you know, because you've experienced what they're going to have so you could in effect order them and we did do, you know, if someone came in with renal colic I would order, you would just do it because that's what you knew the patient was going to have." *(Interview, Nurse, Time 1)

#### Time with patients

The introduction of the new system was perceived to reduce the time healthcare professionals could spend with patients, leaving clinical staff frustrated, as direct patient care was seen as more central to their role and professional identity. The increased time spent in front of the computer thus adversely impacted on job satisfaction.

*"I mean the fact that there's no jobs in the NHS at the moment is the only reason why people would have stayed and morale has been, people are just not feeling job satisfaction because as I say when you should be seeing patients you're actually sitting at a screen that is going interminably slow*." (Interview, Therapist, Time 2)

In some instances, the technology also reduced the perceived quality of the interaction with patients. Using computers whilst consulting was, for example, felt to impact on communication flow, rendering the consultation more formal and less engaging.

*"It means that it's almost like a barrier to communication because you're having to take your eyes off that patient and break that communication to look down at a laptop or a computer and I don't think that's terribly professional..." *(Interview, Allied Healthcare Professional, Time 1).

#### Other systems such as paper

It also became apparent that the introduction of the new system not only impacted on individuals, but also had an effect on paper records (which were, as mentioned above, still used in parallel). Here, paper was often found to be more distributed across geographical locations within healthcare settings. For example, different users would take paper files to their desks at different times to file the electronic print-outs whereas before the introduction of the system all paper records were held centrally in the reception area.

*The paper comes out of a printer in his office but he [Therapist] explains that this printer is also shared, he signs the paper and explains that he "now have to find the paper file to put the paper copy into it", he says that sometimes he cannot find the paper file as other clinicians might have it*... (Researcher Notes, Observation).

As a result, other users needed increasingly to *"chase" *paper records.

*"Oh yes, I mean I've had one where I've said "I'm sure someone has seen this case" and of course it's just not on the file cause it's still with other people. And when it's things like medication and things like that, sometimes I'll just come in and I've not been in for a week and I just need to pick up the file and there is nothing there and I know she's seen them but it's not typed in which never happens with notes cause they just sit there and write it while the client is there. So it's very time consuming." *(Interview, Therapist, Time 2)

#### The medical record itself

Similarly, data entry itself was impacted upon, this in the main manifesting as delays in transcribing the record with the introduction of the new system. This was due to the software being perceived as slow and as impacting on the communication flow with the patient. Notes were therefore often typed up at the end of the day or, in some instances, days after the consultation had taken place. Paper notes were used in the meantime as reminders.

*"Well at the moment because we've sort of piloted it on the [name] wards we have tried to make an effort to use [name of system] as much as possible. I mean we were asked to try and request investigations live on the ward rounds using a portable computer but unfortunately as I said because of constraints of time it wasn't possible to use it that way, it just took too long because we, you know, we have a fair number of patients to see in a short space of time. And so the junior staff are making notes of who needs which investigation and then they're requesting them via [name of system] at the end of the ward round." *(Interview, Consultant, Time 1)

As a result of this delayed data entry, the new computer system was found to be less up to-date than systems that were previously in place.

*He [nurse] then puts the Smartcard [an electronic card used for user identification] into the keyboard and picks up a folder of patient notes on his desk, on top of the folder is a small notepad page with some scribbles on it, he then looks at his paper diary (like a book and full of scribbles) and looks up when he saw the patient, "ah 2 pm on Friday" (it's Monday today), he says that he jots down notes on paper "to jog my memory", it is a girl with an eating disorder*... (Researcher Notes, Observation).

#### Managerial reports

Managerial outputs were also affected by the introduction of the new electronic system. When staff employed workarounds, this would in some cases result in inaccurate reports being generated by the system further down the line, impacting on managers' ability to track activity levels.

*"... so you're getting a certain amount of well I'll just try anything that makes it look like its worked and then you get problems with back-end data because there's a diversification of the numbers of processes that people are using cause they are just desperate to try and get from A to B in a day and they'll try any route to get there that looks like it's working to them*." (Interview, Manager, Time 2)

An interesting example given in this context was that users in outpatients would book appointments on the system using a route that was less laborious, but which meant that, although these appointments would show on their screens as booked, they would not show on the rest of the system as booked. As a result, managerial outputs became so unpredictable that they in a number of instances no longer reflected the reality of what actually happened.

### Conceptualising overall consequences for work practices

**Figure 1:**
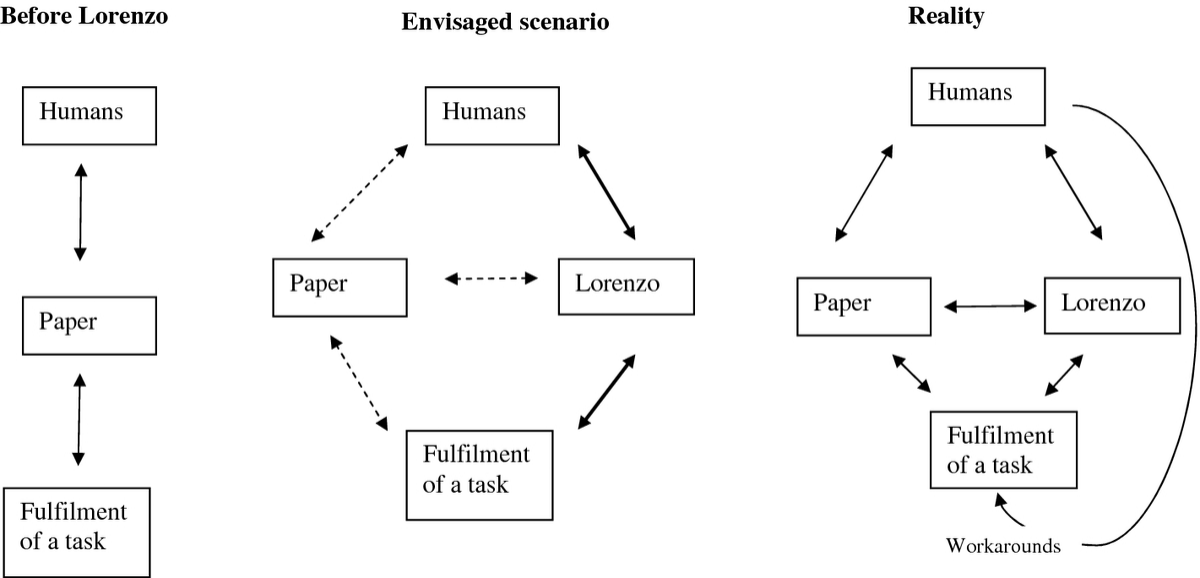
**An ANT-based diagrammatic presentation of the changed networks**.

ANT helped us to conceptualise changes in constellations between various network components including humans, paper and the technology. This is illustrated in Figure 1, which depicts existing work practices before the introduction of Lorenzo, planned changes in work practices with the introduction of Lorenzo, and actual changes in work practices resulting from the introduction of Lorenzo.

## Discussion

### Summary of main findings

The decision to nationally procure EHR systems, coupled with the deliberate decision to limit customisability, resulted in significant changes to user work practices. Users had to adapt to a system that they did not believe was fit for purpose. If they could not resist use, they tended to employ workarounds which were often unanticipated by management. These had several knock-on effects on other factors within the hospital, including hierarchical structures and patterns of communication within the multi-disciplinary healthcare team, the time and quality of interactions with patients, paper records (which became more distributed), the timeliness of the record itself, and managerial outputs (which became less predictable).

### Strengths and limitations of this work

Drawing on a range of data sources has allowed us to triangulate our findings and gain an insight into both formal and informal work practices associated with both intended and unintended workarounds [42]. ANT proved useful in guiding our data collection by placing the technology at the heart of the investigation, helping to appreciate the fluidity of sociotechnical processes, and the active role of the technology on other factors around it, both human and inanimate. It however proved less useful in explaining changes in the technology resulting from the way it was received by users and organisations. Other theoretical lenses may help to address this issue, such as for example the Strong Structuration Theory and the Social Shaping of Technology [40,43], which emphasise the mutually shaping relationship between structures and agents.

Our longitudinal design meant that we were able to explore certain changes and developments over time, but this was still, however, limited to early implementation and adoption. We therefore observed certain ways in which users accommodated the system over time, but despite spending many months in the field we did not have the opportunity to assess the more embedded use of the software. Given our limited funding, we were also unable to develop a detailed understanding of the way healthcare workers used paper systems by examining work practices before the introduction of the new system, although this has often been advocated as an important first step towards understanding how electronic systems are likely to affect individual and collaborative work [42,44-46].

In addition, it has to be kept in mind that: 1) work practices are not static, but constantly changing/adapting (which complicates planning) [47]; and 2) work processes are contingent upon situations [48]. It may be, therefore, that over time and in different contexts users may have developed more (or indeed less) effective ways of using the technology. Consequently, despite our results being likely to be transferable to similar settings (i.e. sites implementing early releases of the same software), they may not apply to all care activities and situations.

### Considering our findings in relation to the wider literature

**Figure 2:**
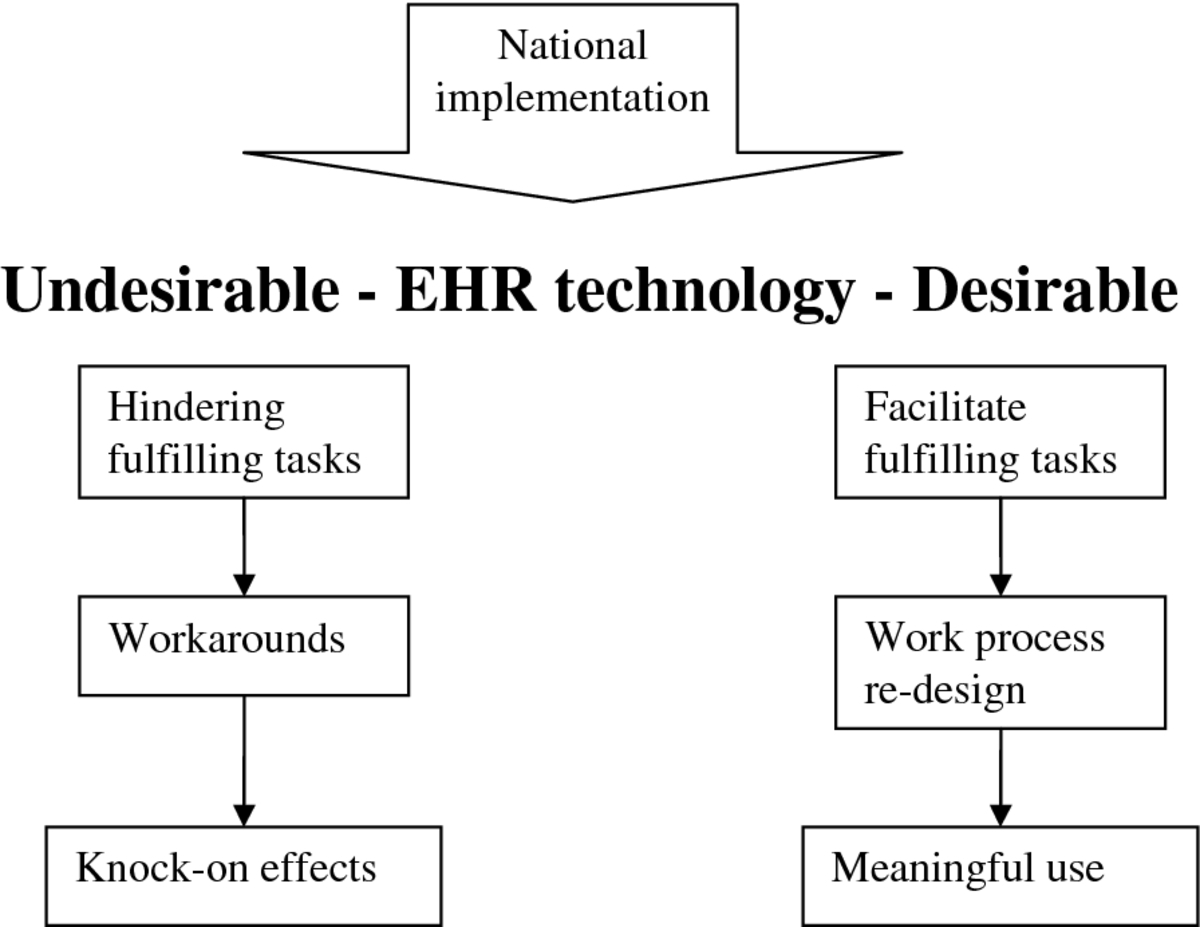
**Diagrammatic presentation of the desirable and undesirable consequences of Lorenzo for user work practices**.

Our results have shed light on the complex consequences and knock-on effects of a nationally procured EHR system for the work practices of a range of users. We illustrate these diagrammatically in Figure 2.

Usability of the system was perceived to be lacking, as the design of the "one-size-fits-all" technology limited tailoring to user needs. It is commonly acknowledged that increased customisation can facilitate the introduction of technology within user work practices [49-53], so the finding that the relatively inflexible nature of Lorenzo resulted in unintended workarounds is probably not surprising [21-23,54].

Users needed to change their work processes to fit in with the technology; and this could be expected with the introduction of a new system as new routines need to be developed and existing work practices inevitably change to some extent [14]. However, issues with poor system usability resulted in clinicians employing informal workarounds to accommodate a system that was viewed as lacking fitness for purpose and increasing workloads and stress levels, as well as taking time away from patient care. When examining the literature on 'workarounds', most of the responses users in our study demonstrated can be thought of as "essential hindrance workarounds". That is, they were used to get around perceived problems in systems that were seen to impact on the more direct task of care provision. Most of the workarounds we identified were also viewed as essential as they were designed to save time that was needed for adequate patient care [22]. However, paradoxically these workarounds, designed to reduce the workload of users, also made the use of the system very specific to users' own contexts, thereby hampering the possibility of meaningful communication between interoperable systems being used in different contexts [55].

We have outlined several knock-on effects of these workarounds. Changes to existing professional responsibilities, hierarchical structures and ways of collaborative working of the multi-disciplinary healthcare team have been found in previous studies investigating the impact of new IT systems in healthcare settings [16,56-59]. Similarly, the finding that new technology can adversely impact on the interactions with patients is reinforcing existing evidence [60]. However, the effects on other factors outside of the immediate environment of use (e.g. organisations and paper records) are indeed new findings, possibly because they are context dependent. These included paper (which became more distributed), managerial outputs (which were perceived to become less accurate), and the timeliness of the record (as data entry was reported as being increasingly delayed). Although previous studies have investigated the potential impact of 'workarounds' on the safety and quality of care, the mechanisms by which effects are observed are likely to vary across settings and may not be transferable to other IT applications [24,61].

Our findings are worrying, considering the potential knock-on implications for the quality and safety of patient care (e.g. if an important record is not available when needed). It is therefore vital that these wider factors are considered when mapping anticipated changes to work practices, which needs to be routinely and regularly done by organisations preparing for changes associated with IT applications in healthcare [62,63].

Considering the range of staff and contexts of IT use in healthcare settings, mapping of work practices needs to be local by definition. Likewise, emerging 'workarounds' are likely to reflect the idiosyncrasies of the individual setting and are therefore highly context-dependent. This is in itself at odds with a national implementation with limited system customisability. Here, work practices may be effectively mapped, but the reality of systems-in-use is likely to vary from planned patterns as informal and emergent work practices that characterise the dynamic and ever-changing hospital environment are often not taken into account [42,64]. This, in turn, may result in a lack of attention being paid to consequences for other stakeholders. The national nature of the implementation means that the resulting mitigating actions are far too slow to be visible in a timely manner to users on the ground, limiting the potential for organisations to flexibly address unanticipated issues [61]. This is supported by the relative successes of small-scale EHR implementations characterised by extensive customisation of systems to suit local needs [65].

In relation to England's national IT strategy, a more participatory approach to development and implementation has repeatedly been advocated [6,66,67]. For example, Catwell and Sheikh have suggested that this should be characterised by early user involvement and development of a shared vision, formative evaluations and testing of prototypes to assess whether the system is perceived as usable, potential re-design so that it fits with users' needs, summative evaluation and benefits identification once implementation completed, and incorporation of issues identified along the way [67].

Engagement and user input in design is clearly important [6,21,66,68-71], but in line with our findings, it is likely to be difficult to realise in the context of a centrally procured system that is intended for implementation on a national scale. This was found to be the case in relation to Lorenzo, which was intended to be "co-creational" by involving users in system design. However, the result of the focus on interoperability was limited local input and a resulting application that did in many instances not fulfill user and organisational needs. In addition, the organic development of engagement was hampered from the start as organisations and users were mandated to implement and use Lorenzo. Our experiences and the wider evidence-base suggest that a potential way forward may involve implementing a combination of standardised core solutions and customised elements, corresponding to particular users and environments (also known as 'configurational technology') [72,73].

### Implications for policy, practice and research

Our findings provide important insights relating to the impact of nationally procured systems on both formal and informal work practices of users and collaborative working. There is clearly a need to understand better how coordination is achieved without IT in order to produce a system that has the ability to facilitate this coordination or indeed revolutionise it by making processes more effective [42]. This is likely to vary across different settings and will warrant detailed small-scale local studies over longer periods of time in order to trace how work is structured with paper systems, as well as the initial, continuing and more embedded use of new EHR systems. As part of this work, it may be helpful to explore these issues from a range of theoretical angles. These may include deliberations surrounding the classification of an EHR system as a medical device and associated considerations surrounding usability testing including assessments of potentially negative consequences; more specific discussion surrounding effective leadership in organisations that have a high degree of autonomy; and exploration of ways in which evidence-based practice can be effectively promoted and integrated within individual workflows and within political strategies.

With regards to the impact on the quality and safety of care, there is a need to assess outcomes identified in our study quantitatively, in order to determine whether the mechanisms identified are indeed contributing to adverse patient outcomes. We do, however, acknowledge that such studies will be challenging to design as outcome measures may not necessarily be traceable to the system and comparators may be difficult to identify [74,75].

The implications for practice emerging from our research are two-fold: firstly, there is a need to allow local work process mapping and responses to emergent practices and perceived inadequacies in the technological design; and secondly, it is vital not only to assess workarounds employed by local users, but the reasons behind their adoption in order to be in a position to reflect on other downstream consequences.

## Conclusions

We have identified a number of important consequences resulting from key procurement, design and implementation decision in relation to plans to implement a national EHR software system in England. These consequences were observed in relation to healthcare staff, organisations, medical records and patients.

We have furthermore highlighted that assessments of workarounds and their consequences need to be locally undertaken and addressed. In the context of the particular implementation investigated in our work, this was inhibited by the focus on interoperability at the expense of customisability in national efforts. The question surrounding potential trade-offs between large-scale interoperability and local requirements is likely to be the subject of continuous debate in England and beyond with no easy answers in sight. Based on the findings from this study, we suggest that concerns relating to interoperability should not be prioritised over-and-above ensuring that essential local needs are met. This is because a failure to address these local needs not only risks the viability of the entire implementation effort, but it may also severely hinder successful adoption which may result in important adverse local consequences (as we have shown). In addition, unintended use of local systems may also impact on interoperability considerations in relation to sharing of data between different healthcare providers. This may be the case if data held within local systems does not accurately reflect reality, potentially resulting in inaccurate information being drawn on by other providers.

Informal work practices, and knock-on effects are expected to vary, but assessing and tracing changes and effects over time is likely to pay significant returns for the implementing organisation, users and the quality of patient care.

## Competing interests

The authors declare that they have no competing interests.

## Authors' contributions

KC is guarantor. AS and AW contributed to interpreting the evidence and drafting the manuscript. KC collected and analysed the data, and led the drafting of this paper. All authors read and approved the final manuscript.

## Pre-publication history

The pre-publication history for this paper can be accessed here:

http://www.biomedcentral.com/1472-6947/12/15/prepub
